# Comparison of six different methods to calculate cell densities

**DOI:** 10.1186/s13007-018-0297-4

**Published:** 2018-04-16

**Authors:** Carolina Camacho-Fernández, David Hervás, Alba Rivas-Sendra, Mª Pilar Marín, Jose M. Seguí-Simarro

**Affiliations:** 1 COMAV - Universitat Politècnica de València, CPI, Edificio 8E - Escalera I, Camino de Vera, s/n, 46022 Valencia, Spain; 2 Biostatistics Unit – IIS La Fe, Valencia, Spain; 3 Microscopy Unit – IIS La Fe, Valencia, Spain; 4 Present Address: Universidad Regional Amazónica IKIAM, Tena, Ecuador

**Keywords:** Automated cell counter, Cell counting, Flow cytometry, Fluorospheres, Hemacytometer, Image analysis, Microscopy, Microspore culture

## Abstract

**Background:**

For in vitro culture of plant and animal cells, one of the critical steps is to adjust the initial cell density. A typical example of this is isolated microspore culture, where specific cell densities have been determined for different species. Out of these ranges, microspore growth is not induced, or is severely reduced. A similar situation occurs in many other plant and animal cell culture systems. Traditionally, researchers have used counting chambers (hemacytometers) to calculate cell densities, but little is still known about their technical advantages. In addition, much less information is available about other, alternative methods. In this work, using isolated eggplant microspore cultures and fluorescent beads (fluorospheres) as experimental systems, we performed a comprehensive comparison of six methods to calculate cell densities: (1) a Neubauer improved hemacytometer, (2) an automated cell counter, (3) a manual-counting method, and three flow cytometry methods based on (4) autofluorescence, (5) propidium iodide staining, and (6) side scattered light (SSC).

**Results:**

Our results show that from a technical perspective, hemacytometers are the most reasonable option for cell counting, which may explain their widely spread use. Automated cell counters represent a good compromise between precision and affordability, although with limited accuracy. Finally, the methods based on flow cytometry were, by far, the best in terms of reproducibility and agreement between them, but they showed deficient accuracy and precision.

**Conclusions:**

Together, our results show a thorough technical evaluation of each counting method, provide unambiguous arguments to decide which one is the most convenient for the particular case of each laboratory, and in general, shed light into the best way to determine cell densities for in vitro cell cultures. They may have an impact in such a practice not only in the context of microspore culture, but also in any other plant cell culture procedure, or in any process involving particle counting.

**Electronic supplementary material:**

The online version of this article (10.1186/s13007-018-0297-4) contains supplementary material, which is available to authorized users.

## Background

Successful in vitro cell cultures depend upon a proper cell density at the onset of the culture. A correct calculation of cell plating density is a critical step for cell cultures, including somatic plant cells [1], protoplasts [2] and microspore cultures. In isolated microspore cultures there is a minimum plating density below which, no embryogenic response is observed [3–5]. On the other hand, microspore densities higher than optimal may inhibit the embryogenic response [6] and reduce the number of viable embryos due to the reduced availability of nutrients and/or to the presence of inhibitory toxins generated by the microspores [3, 5, 7, 8]. Each species has its own optimal culture density. Thus, an optimal plating density was established at 4 × 10^4^ microspores/ml for *Brassica napus* microspore cultures [6], whereas for pepper it was proposed to be 8 × 10^4^–10 × 10^4^ [3], 8 × 10^4^ for rice [9], 12.5 × 10^4^ for rye [10], and even 140 × 10^4^ for apple [11].

A survey among 400 researchers working with animal cell cultures [12] revealed that 71% used counting chambers, also known as hemacytometers, to perform cell density calculations. In isolated microspore cultures, the vast majority of the (few) papers that mentioned the system used to calculate microspore density, referred the use of hemacytometers. Among them, the most used by far is the Neubauer Improved [5, 13–16, for some examples], although others such as the Fuch-Rosenthal [17] or the Burker chamber [18, 19] have been occasionally used. Although scarce, there are also studies that use estimations based on the volume of the pelleted microspores [20], broad assumptions like “X buds (or anthers) equals to Y microspores” [4, 21], or density units of the type “a ratio of X buds/ml” [22] or “X Petri dishes containing microspores isolated from Y flower buds” [23]. The use of other counting methods, not based on counting chambers, has been negligible. Indeed, fast, easy and accurate alternatives such as flow cytometry [24] have not been used for microspore cultures. As far as we know, flow cytometry has only been used to identify populations of embryogenic and non-embryogenic microspores [25] and to identify cellulose deposition in embryogenic microspores [26]. Interestingly, these studies did not use this method for the initial adjustment of microspore density. Other methods based on automated cell counting have not been described in the literature.

Considering the importance of an accurate calculation of the initial microspore density and in general, of cultured cell density, it is surprising that, to our knowledge, comparative studies of the different methods available for this are still very scarce. In this work, we developed a comparative study to determine the accuracy, precision, reproducibility, and in summary, the reliability of six different methods to calculate cell densities, including the most popular (the hemacytometer), but also others, less used but potentially useful as well. We compared the following methods: (1) hemacytometer, (2) automated cell counting, (3) a manual-counting method based on counting microscope fields, (4) flow cytometry-based detection of microspore autofluorescence, (5) flow cytometry-based detection of propidium iodide (PI)-stained microspores, and (6) flow cytometry-based detection of side scattered light (SSC). To test these methods, we used isolated microspore cultures of eggplant, a well-established system in our laboratory [27–30], and also fluorospheres, fluorescent spherical beads certified to be at a known concentration and therefore used as standards for absolute counts. Our results show remarkable differences between the performance of the different methods, supporting the use of some of them, and discouraging the use of others.

## Methods

### Plant materials, microspore culture, and fluorospheres

As donors of eggplant microspores, we used cv. Ecavi (a F1 hybrid from Rijk Zwaan) and the doubled haploid lines DH5, DH29, DH36 and DH40, produced in our laboratory [30] from cv. Bandera (a F1 hybrid from Seminis). Plants were grown in 30 cm pots at COMAV greenhouses (Universitat Politècnica de València) set up at 20 °C under natural light. Flower buds at the appropriate stage of development [31] were immediately transported to the laminar flowhood under melting ice, and processed as previously described [27]. Unless otherwise stated, the estimation of the initial culture density was carried out using an Improved Neubauer Chamber.

We also used fluorescent microspheres (Flow-Count Fluorospheres, Ref. 7547053, from Beckman Coulter), typically used as internal controls for counting cell populations. Fluorospheres are excitable at 488 nm, emit in a range between 525 and 700 nm, and are uniform in size (~ 10 µm) and fluorescence intensity. According to manufacturer’s specifications, the assayed concentration was 1,030,000 fluorospheres/ml. From this batch, different 1:1 (undiluted), 1:2 and 1:10 dilutions in distilled water (containing 1,030,000, 515,000 and 103,000 fluorospheres/ml respectively) were prepared for each different round of measurements with the different methods used.

In order to compare direct and indirect estimation of microspore densities by fluorosphere counting, mixed samples of microspores and fluorospheres were prepared, including 100 µl of fluorospheres in 1 ml of microspore suspension.

### Pipetting technique verification procedure

In order to verify the accuracy of pipetting and to rule out pipetting errors in all the pipetting steps performed in this work, we used the pipetting technique verification procedure described in the Flow-Count Fluorosphere specifications provided by the manufacturer (www.beckmancoulter.com). Basically, this procedure consists of placing a test tube and a weighting vessel on an analytical balance and, after taring the balance, pipetting 100 μl of sample into the test tube and recording the weight. Then, the balance is tared again, and the procedure is fully repeated 10 times with 10 different 100 μl samples. For each pipette, the mean, standard deviation and percent coefficient of variation (%CV) of all the repeats was calculated. According to manufacturer standards, pipettes are well calibrated when the %CV is ≤ 2.0% and the weight measured is the expected for the volume used.

### Field counting with microscope images

We also used a manual-counting method based on a user counting cells in microscope fields. For field counting, 10 images of each microspore culture and fluorosphere dish were taken in a light microscope with a 20 × objective. To ensure randomness for each image, we used a computer program [32] to generate random paths along the dish, which provides different, random-generated coordinates for each field to be imaged. For each image, the total number of particles present in the image was counted, and the average of the 10 images was calculated. In mixed samples, fluorospheres and microspores were individually counted. To avoid inter-counter variation, all countings were performed by the same operator. To calculate particle concentration, the depth of the culture medium on the dish (1.41 mm) was obtained dividing the volume inoculated to the culture dish (3 cm^3^) by the area of the culture dish (21.24 cm^2^). Then, the volume of culture corresponding to the area imaged in the 10 micrographs used (3.1 mm^3^, ~ 3 μl) was obtained by multiplying the culture depth (1.41 mm), by the area of the 10 micrographs (0.22 mm^2^ × 10 = 2.2 mm^2^). For fluorosphere counting, the different dilutions were poured into 3 cm-wide culture dishes. Procedures and calculations were identical to those used for microspore cultures, but using a depth of 1.24 mm.

### Automated cell counting

We used the Micro Counter 3100 system from Celeromics (Grenoble, France). This system is based on the use of an inverted microscope-coupled digital camera to take sets of images of cultured cells or particles within sealed culture dishes. Then, an image analysis algorithm automatically identifies and counts the particles present in images, and the system calculates their density per area and volume unit, according to the parameters previously established during system calibration. For calibration, the culture depth previously calculated was used. For each counted dish, 10 different 20 × images were taken. A computer program [32] was used to generate random paths along which images were taken.

For this procedure (hereinafter referred to as the Neubauer method), we used a Neubauer Improved glass counting chamber (Electron Microscopy Sciences, Hatfield, PA) with a grid of perpendicular lines etched in the middle region (Additional file 1: Figure S1). To allow for comparisons between automated, field and Neubauer counting, we made the three counting efforts similar by adjusting the number of Neubauer cells to be counted. With automated and field counting, a total volume of 3.1 mm^3^ of microspore culture and a similar volume of fluorosphere suspension was counted (10 images × 0.22 mm^2^ of area for each image × the calculated depth, 1.41 mm). To count a similar volume with a Neubauer chamber, we calculated the number of Neubauer large squares (Additional file 1: Figure S1) to be counted by dividing 3.1 mm^3^ by 0.1 mm^3^, which is the volume loaded to each Neubauer large square. The result (31 large squares) was approximated to 30, which corresponded to counting all the microspores present in five large squares of the two cells of the chamber, and repeating this three times (5 squares × 2 cells × 3 chambers = 30). To fill each chamber cell, the content of each culture dish was poured into a 15 ml conical flask. Immediately after a thorough resuspension of microspores or fluorospheres, 10 µl were pipetted out and loaded into the chamber cells by capillarity until they were entirely filled, discarding the unloaded volume. Counting was performed under an inverted microscope with a 10× objective. In mixed samples, fluorospheres and microspores were individually counted. To avoid inter-counter variation, all counting was performed by the same operator.

### Particle counting by flow cytometry

Flow cytometry allows for the fast detection and quantification of fluorescent particles when they are excited with a fluorescent light source. Usually, cells are stained with a fluorescent dye to make them fluorescent. We used three flow cytometry methods based on the detection of (1) microspore autofluorescence, (2) propidium iodide (PI)-stained microspores, and (3) side scattered light (SSC), which is proportional to the overall size, granularity and internal complexity of the measured particle [33] with no need for fluorescence emission. We used a Partec CyFlow Ploidy Analyser (Partec, Gürlitz, Germany) equipped with a UV LED lamp (365 nm), a Nd-YAG green laser at 532 nm (30 mW), and filters for PI (long pass filter 590 nm) and for DAPI (long pass filter 435 nm). For direct counting of unstained microspores, the contents of culture dishes were resuspended and loaded into plastic vials and directly charged into the loading port. For autofluorescence detection (hereinafter referred to as the FC-unstained method), a UV LED lamp and DAPI excitation/emission filters were used. The suspension was loaded in the flow cytometer, which provided the number of fluorescent counts recorded, their fluorescent intensity, and the volume where the counts were recorded. For PI fluorescence-based flow cytometry (hereinafter referred to as the FC + PI method), the Nd-YAG green laser and the PI excitation/emission filters were used. For cell counting, 1 ml of microspore suspension was taken from each culture dish. The suspension was incubated with 0.5 ml of Partec nuclei extraction buffer during 60 s, and then with 2 ml of Partec staining buffer + 12 µl PI for 1 h at 4 °C. The stained suspension was loaded in the flow cytometer, which provided the number of fluorescent counts recorded, their fluorescent intensity, and the volume where the counts were recorded. For fluorosphere counting, 500 µl of 1:1 (undiluted), 1:2 and 1:10 fluorosphere dilutions were placed in plastic vials. Additional 500 µl of water were added for a final loading volume of 1 ml of 1:2, 1:4 or 1:20 dilutions, respectively. The data obtained were multiplied by 2 to be comparable with those of other methods. Vials were directly charged into the loading port. To calculate particle density based on the SSC emission (the SSC method), the flow cytometer provided the number of SSC counts recorded, their SSC intensity, and the volume where the counts were recorded. For SSC, the Nd-YAG green laser and the PI excitation/emission filters were used. To test to what extent sedimentation of microspores at the bottom of the vial, where the aspiration system cannot reach, could be reducing the number of counted microspores, we counted the cells suspended in the vial, washed the vial thrice with one additional ml of culture medium to resuspend the potentially sedimented microspores, counted the cells of each washing medium, and then calculated the density by dividing the total number of microspores counted in all rounds (including washings) by the initial volume where microspores were suspended (excluding washings). For all flow cytometry-based methods and cultures, three different samples were taken and processed, their individual densities calculated and then averaged. For all methods, particle densities were calculated dividing the total number of counts by the volume loaded for these counts. In mixed samples, fluorosphere and microspore densities were individually calculated using SSC counts from all the peaks obtained. After each counting round, the whole system was thoroughly cleaned to prevent wrong counts from previous samples.

### Parameters used and statistical analysis

Based on the definitions of the International Organization of Standardization (ISO, www.iso.org; Additional file 2: Table S1), we calculated the following parameters for the comparisons described in this work:

- Precision: the closeness between independent test results under the same conditions.
- Accuracy: the closeness of agreement between the data and the real or reference value.
- Reproducibility: the similarity of data obtained with the same method from samples in different conditions.

In addition, we defined concordance as the agreement of measurements obtained with different methods.

Precision was assessed by analyzing the dispersion of data obtained in repeated measures of the microspore/fluorosphere suspensions with the same initial density with the same method. Accuracy was assessed by calculating the percentage of deviation of each individual measurement from the theoretical (for microspores) or real value (for fluorospheres). For a visual and easy-to-understand representation of accuracy and precision results, box and whiskers graphs were developed for each method and group of samples. In all cases, repeated measurements were summarized by their mean and standard deviation. To assess the reproducibility of each method, two different measurements were performed in the same cultures, the first at days 3 and the second at days 18. Reproducibility was assessed by Bland–Altman plots comparing both measurements, computing their coefficient of repeatability (CR) and performing an ANOVA analysis. The average bias and 95% limits of agreement were also computed for each plot. Concordance among methods was assessed using pairwise Bland–Altman plots [34]. As alternative concordance estimation, Lin’s concordance correlation coefficient [35] was also computed in each case. Statistical analysis and charts were performed with the R software (version 3.1.1) [36].

## Results

**Fig. 1:**
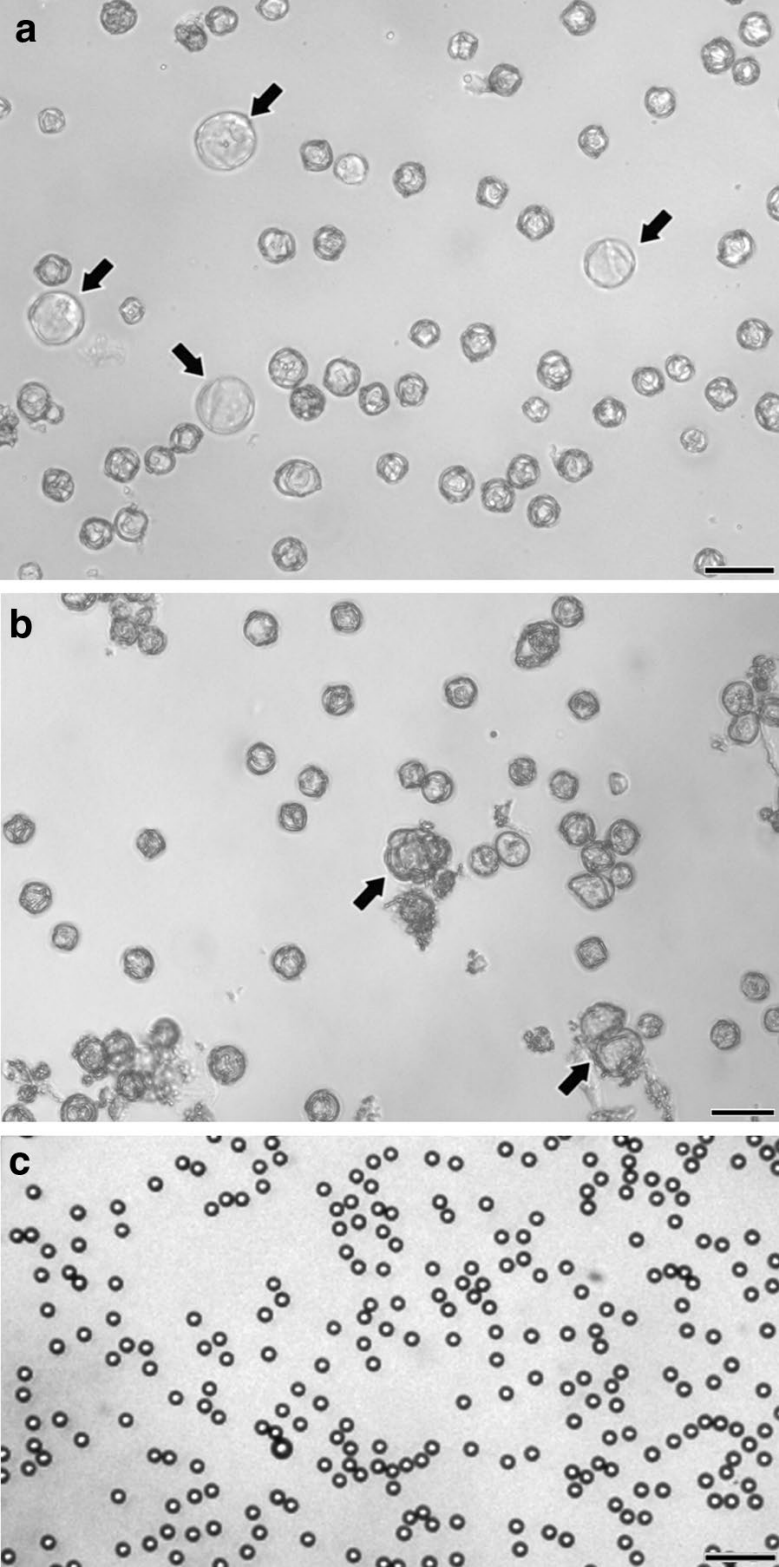
**a** Three day-old eggplant microspore culture. Cultures at this stage are principally composed of regular eggplant microspores together with few slightly enlarged microspores (arrows). **b** Eighteen day-old eggplant microspore culture. Cultures at this stage are principally composed of regular eggplant microspores together with few enlarged microspores or microspore-derived embryos (arrows). **c** Fluorospheres. These particles are very regular in size and shape. Bars: 50 µm

For evaluation of precision, accuracy, reproducibility and concordance between methods, a total of 17 microspore cultures were performed and measured using the different methods tested in this work. For each of these, the initial density was adjusted to 400,000 microspores/ml using the Neubauer method as described in *Methods*. Microspore density was estimated for each culture at days 3 and 18. 3 day-old cultures are formed by microspores induced towards embryogenesis, together with non-induced (pollen-like), dead and arrested individuals. However, this culture stage is still too early to detect important morphological differences between them. Thus, in terms of morphology, cultures at this stage are characterized by the presence of regular eggplant microspores, identical to those present in the anther, and microspores swollen as a consequence of the androgenic switch that makes them to enlarge within the exine (arrows in Fig. 1a). In turn, 18 day-old cultures present enlarged microspores or microspore-derived embryos, produced as a consequence of cell divisions within the exine (arrows in Fig. 1b). However, most of the cultured structures are arrested and/or dead microspores, or pollen-like structures. These different developmental fates imply size increases only in embryogenic and pollen-like microspores, but the total number initially inoculated in culture dishes remains unchanged. In turn, suspended fluorospheres (Fig. 1c) look as isolated, uniform particles dispersed in the culture.

### Precision and accuracy of methods

As a preliminary step to be confident with all the analysis performed, we checked the precision of the pipettes used in this work. The pipetting verification procedure described in *Materials and methods* yielded %CVs of 0.6 and 0.08% for the 10–100 µl and the 100–1000 µl pipette, respectively. These %CVs are notably below the 2% threshold established as a reference by the manufacturer. Therefore, we assumed that our pipettes were well calibrated, and therefore valid for this study.

**Fig. 2:**
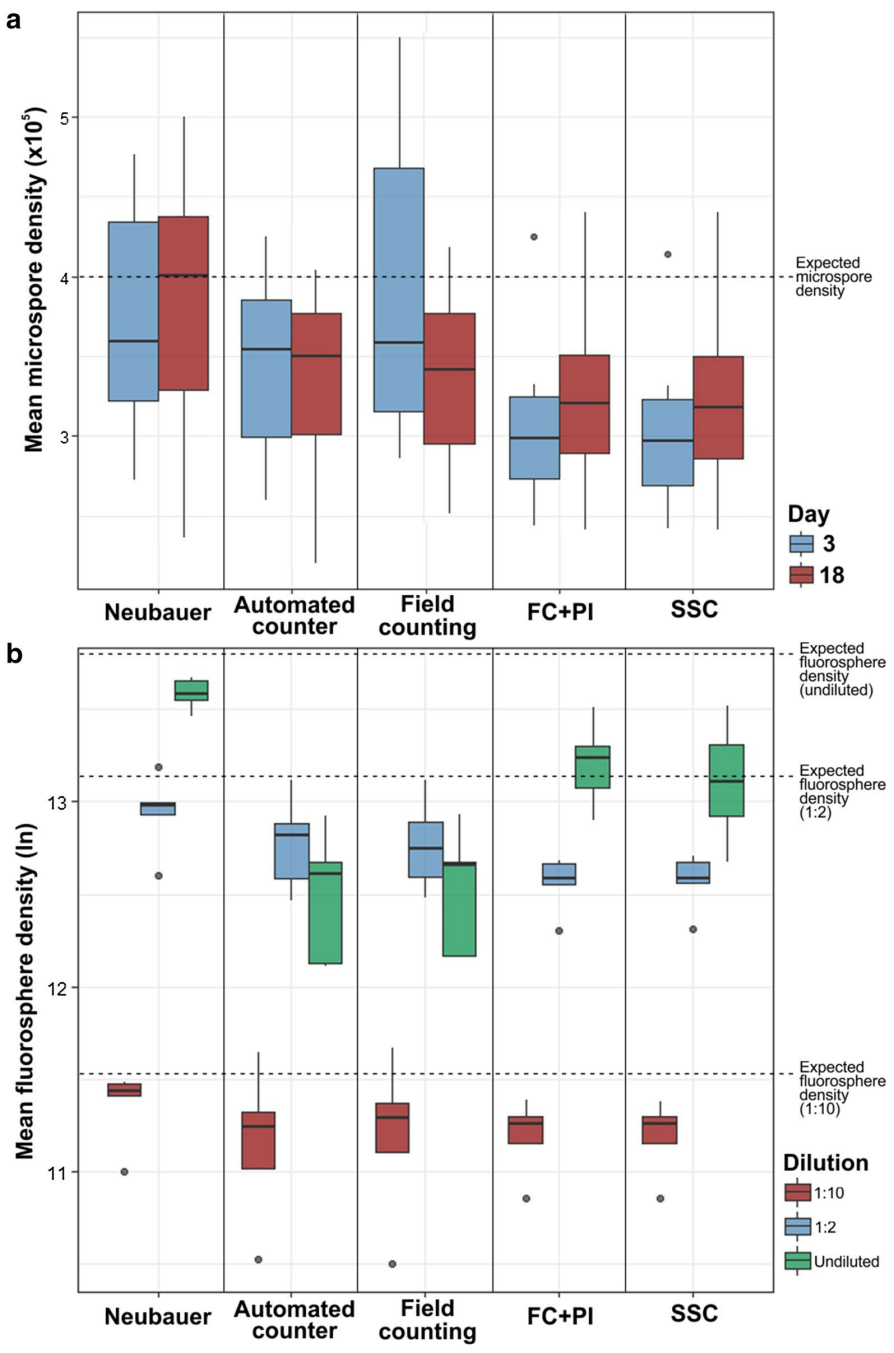
Box-and-whiskers plots for **a** mean densities of 17 different eggplant microspore cultures measured at days 3 and 18, **b** fluorospheres at three different concentrations: 1:10, 1:2 and 1:1 (undiluted, 1,030,000 microspores/ml). Dashed lines represent the expected microspore (in **a**) and fluorosphere densities (in **b**). Note that values in B are expressed as neperian logarithms of mean fluorosphere densities. See text for further details

We calculated the mean, standard deviation, median and 1st and 3rd quartiles of measurements performed with each method (Table 1). Figure 2a shows a graphical representation of the measurements in cultures at days 3 and 18. Next, we performed five independent measurements of fluorosphere suspensions with each method, using the undiluted, 1:2 and 1:10 dilutions. Results are shown in Table 2 and represented in Fig. 2b. For both microspore cultures and fluorosphere suspensions, counts with the Neubauer chamber were in general higher than with all other methods, deviating up to ~ 50% in average from the rest of methods in the case of fluorospheres. All the means were below the theoretical or real density. In terms of precision, the methods based on flow cytometry showed the highest values (lowest dispersion) and the Neubauer method showed the highest dispersion of data in microspore measurements (Fig. 2). Nevertheless, the Neubauer method showed a high precision in fluorosphere measurements. The precision of methods that count cells from images taken from the dish (automated and manual-counting methods) presented the lowest values. In terms of accuracy, the Neubauer chamber presented the highest means, therefore closest to the expected values (~ 13% below in average). Results were remarkably closer to the assumed reference value when undiluted suspensions were measured with the Neubauer method, deviating only 22.5%. However, all other methods were markedly below, with an average difference of ~ 42%, indicating a low accuracy. In undiluted suspensions, some methods seemed to deviate from the expected value more than at higher dilutions, suggesting that their accuracy may depend on particle density (Fig. 2b). That was the case of image-dependent methods (manual-counting and automatic counter methods).

**Table 1:** Mean and standard deviation (first row), median and 1st–3rd quartile (second row), and percentage of deviation from the initial value and coefficient of variation expressed in percentage (third row) of each counting method in microspore cultures at days 3 and 18

Method	Days 3	Days 18
Neubauer	371.8 (65.2)	383.3 (79.3)
	359.4 (322.2–434.2)	400.8 (328.6–437.2)
	7.1/17.5%	4.2/20.7%
FC unstained	220.3 (90.1)	255.2 (108.4)
	197.8 (173.7–247.6)	255.2 (216.9–293.6)
	44.9/40.9%	36.2/42.5%
Flow cytometry + PI	309.0 (60.2)	332.0 (60.5)
	298.8 (273.3–324.6)	320.6 (289.2–350.4)
	22.8/19.5%	17.0/18.2%
Flow cytometry + SSC	305.4 (57.4)	329.6 (60.5)
	296.5 (269.2–323.3)	317.8 (285.9–350.2)
	23.7/18.8%	17.6/18.4%
Automated counter	349.2 (50.3)	335.5 (52.9)
	354.0 (299.0–385.5)	349.5 (301.2–376.5)
	12.7/14.4%	16.0/15.9%
Field counting	386.9 (81.5)	336.4 (53.9)
	358.1 (315.8–467.9)	341.4 (294.9–377.2)
	3.3/21.1%	15.9/16.0%

Values are expressed in thousands of microspores/ml

**Table 2:** Mean and standard deviation (first row), median and 1st–3rd quartile (second row), and percentage of deviation from the initial value and coefficient of variation expressed in percentage (third row) of each counting method and dilution of fluorosphere suspensions

Method	1/10 dilution	1/2 dilution	Undiluted
Neubauer	87.2 (15.5)	423.3 (84.3)	797.9 (67.8)
	92.7 (90.0–96.3)	432.3 (412.7–440.0)	795.0 (769.7–854.7)
	15.3/17.8%	17.8/29.9%	22.5/8.5%
Flow cytometry + PI	73.5 (13.8)	287.2 (40.4)	554.6 (128.3)
	77.3 (69.8–80.7)	292.7 (283.4–317.6)	559.0 (477.4–599.4)
	28.7/18.8%	44.2/14.1%	46.2/23.1%
Flow cytometry + SSC	73.4 (13.8)	289.5 (42.7)	513.7 (166.1)
	77.2 (69.7–80.6)	293.3 (284.8–318.6)	491.2 (411.1–602.1)
	28.7/18.8%	43.8/14.7%	50.1/32.3%
Automated counter	74.1 (28.4)	361.8 (92.6)	278.6 (96.6)
	76.1 (60.7–82.4)	369.5 (291.7–392.1)	299.1 (184.8–318.6)
	28.1/38.3%	29.7/25.6%	73.0/34.7%
Field counting	77.2 (29.2)	359.2 (91.9)	286.2 (95.1)
	79.9 (66.4–86.9)	343.9 (295.2–397.1)	315.1 (192.2–318.6)
	25.0/37.8%	30.3/25.6%	72.2/33.2%
Real value	103.0	515.0	1030.0

Values are expressed in thousands of fluorospheres/ml

The use of flow cytometry to measure autofluorescence of unstained microspores was the method that showed the worst performance for all parameters tested (Table 1). In an attempt to find out the cause of such a discrepancy, we speculated that it could be due to sedimentation of microspores at the bottom of the vial, where the aspiration system cannot reach. To test this, we designed a set of experiments (see “Methods” section) consisting of recovering the potentially sedimented microspores through successive washing rounds. However, the results of these experiments were not different from those without washings (data not shown). Therefore, we concluded that microspore autofluorescence was not sufficiently high or homogeneous to detect all the microspores passed through the flow cytometer. As a consequence, we decided to discard flow cytometry with unstained microspores for further experiments.

### Reproducibility of methods

**Fig. 3:**
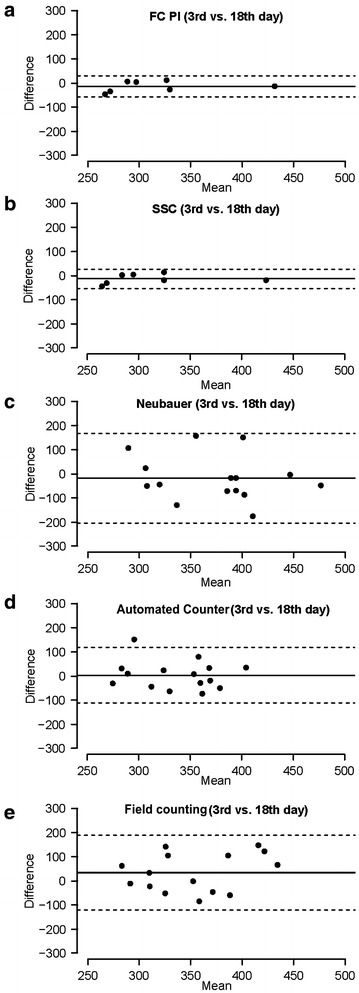
Bland–Altman comparisons of reproducibility of each method by comparing 3 and 18 day-old microspore culture data. Difference values of the Y axis are expressed in thousands

For the analysis of the reproducibility of methods, we compared measurements in samples at two different moments of culture progression (days 3 and 18). Differences between measurements for each method are depicted by Bland–Altman plots in Fig. 3. As a formal measure, the coefficient of repeatability (CR) was obtained for each method (Table 3) using data of these two different conditions. Moreover, we performed an ANOVA analysis comparing data from both days 3 and 18 where none of the methods showed significant differences (Table 3). Our results showed that both FC + PI (Fig. 3a) and SSC methods (Fig. 3b) were the most reproducible, showing narrow limits of agreement and the lowest CR values (Table 3). The Neubauer method (Fig. 3c) and the automated counter (Fig. 3d) appeared as moderately reproducible, as they presented wider limits of agreement and higher CR values (Table 3). Finally, field counting (Fig. 3e) was the least reproducible method. It showed a high CR, the widest limits of agreement, and a bias (~ 35 above) much higher than other methods, where bias ranged between − 17 and 3.

**Table 3:** Assessment of the repeatability and reproducibility of each of the methods tested, expressed by a coefficient of repeatability (CR) and *p* value of ANOVA analysis comparing days 3 and days 18

Method	CR (95% Cl)		p-value ANOVA
Neubauer chamber	175.9	(100.6, 233)	0.4976
Flow cytometry + PI	39.2	(18.1, 53.6)	0.6597
Flow cytometry + SSC	36.3	(19.5, 50.8)	0.6652
Automated counter	109.6	(66.7, 156.8)	0.8236
Field counting	148.4	(113.3, 177.5)	0.1479

Bracketed numbers represent the 95% confidence limits (Cl)

### Concordance between methods

**Fig. 4:**
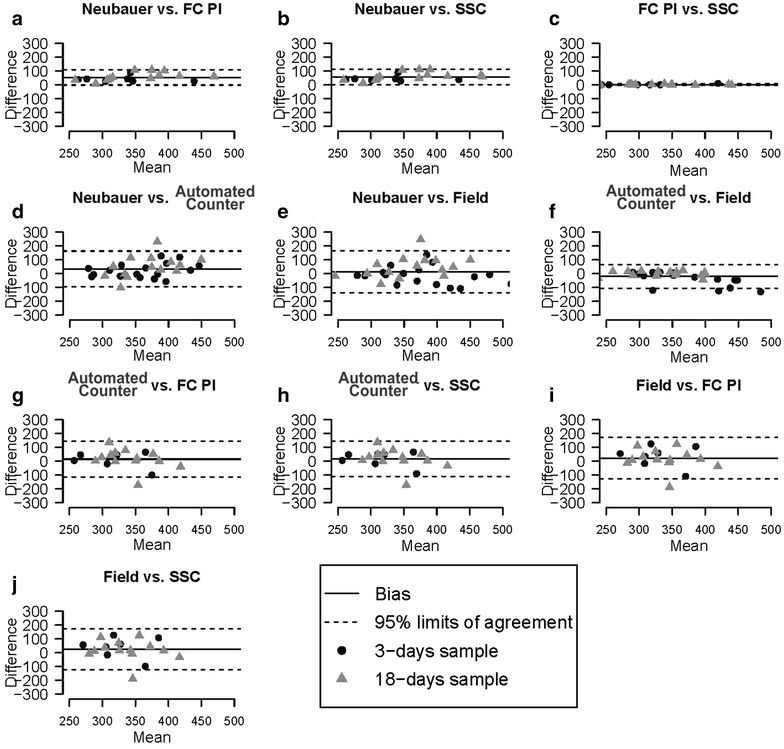
Bland–Altman comparisons of agreement between methods. Difference values of the Y axis are expressed in thousands

In order to analyze the level of agreement between methods, we performed Bland–Altman plots from microspore density measurements (Fig. 4). The lowest limits of agreement appeared when Neubauer, SSC and FC + PI methods were compared between them (Fig. 4a–c). SSC and FC + PI (Fig. 4c) showed the highest level of agreement, as revealed by the minimal limits of agreement and non significant bias of their comparisons. However, comparisons between Neubauer and cytometry-based methods (Fig. 4a, b) presented a very high bias. Thus, despite their good level of agreement, flow cytometry methods seem to induce a non-negligible underestimation, at least, with respect to Neubauer method. In general, automated counter and field counting methods showed low concordance with the rest of methods (Fig. 4d–j), presenting the highest bias when compared to the Neubauer method (Fig. 4d, e), and wide limits of agreement with all methods except between themselves (Fig. 4f). Fluorosphere measurements were not used for this comparison because they have a known, real value to compare with.

Additionally, we assessed agreement between all methods using Lin’s concordance correlation coefficient (Table 4). For comparisons between methods, the Lin’s concordance correlation coefficient is preferred over the standard Pearson correlation coefficient because a Pearson correlation does not detect constant biases, yielding perfect correlations between remarkably differing methods that have a constant bias. On the other hand, with the Lin’s concordance correlation coefficient it is possible to detect the presence of constant bias. Using it, we obtained results similar to those of the Bland–Altman plots. In general, microspore counts showed in all cases values higher than fluorosphere counts. Concordances between methods were minimal when high fluorosphere concentrations were used, with concordance coefficient values near to zero. The best levels of agreement were found in comparisons between flow cytometry-based methods (concordance coefficient ranging from 0.7 to 1.0) and between field and automatic counter (from 0.5 to 1). The Neubauer method showed moderate concordance values when compared to flow cytometry methods (from 0.5 to 0.8) except for high fluorosphere concentrations. The lowest level of concordance in microspore counts was found when the field counting method was compared with the Neubauer, PI and SSC.

**Table 4:** Agreement between methods using Lin’s concordance correlation coefficient

Comparison	Lin’s concordance correlation coefficient				
	Microspore cultures		Fluorosphere suspensions		
	Days 3	Days 18	Undiluted	1:2 dilution	1:10 dilution
Neubauer					
Flow cytometry + PI	***0***.***75***	0.52	*0*.*01*	*0*.*01*	***0***.***63***
Flow cytometry + SSC	***0***.***72***	0.49	*0*.*04*	*0*.*01*	***0***.***63***
Automated counter	*0*.*35*	0.45	*0*.*00*	*0*.*22*	*0*.*07*
Field counting	− *0*.*01*	0.52	− *0*.*01*	*0*.*21*	− *0*.*04*
Flow cytometry + PI					
Flow cytometry + SSC	***1***.***00***	***1***.***00***	***0***.***69***	***1***.***00***	***1***.***00***
Automated counter	0.40	0.64	*0*.*23*	− *0*.*20*	*0*.*22*
Field counting	− *0*.*01*	0.52	*0*.*24*	− *0*.*14*	*0*.*10*
Flow cytometry + SSC					
Automated counter	0.41	0.62	*0*.*20*	− *0*.*22*	*0*.*22*
Field counting	*0*.*01*	0.51	*0*.*21*	− *0*.*16*	*0*.*10*
Automated counter					
Field counting	0.48	***0***.***88***	***0***.***99***	***0***.***99***	***0***.***98***

See text for further details. High, moderate and low agreement values are represented in bolditalic, regular and italics characters, respectively

### Adjustment of the initial density with different counting methods

**Fig. 5:**
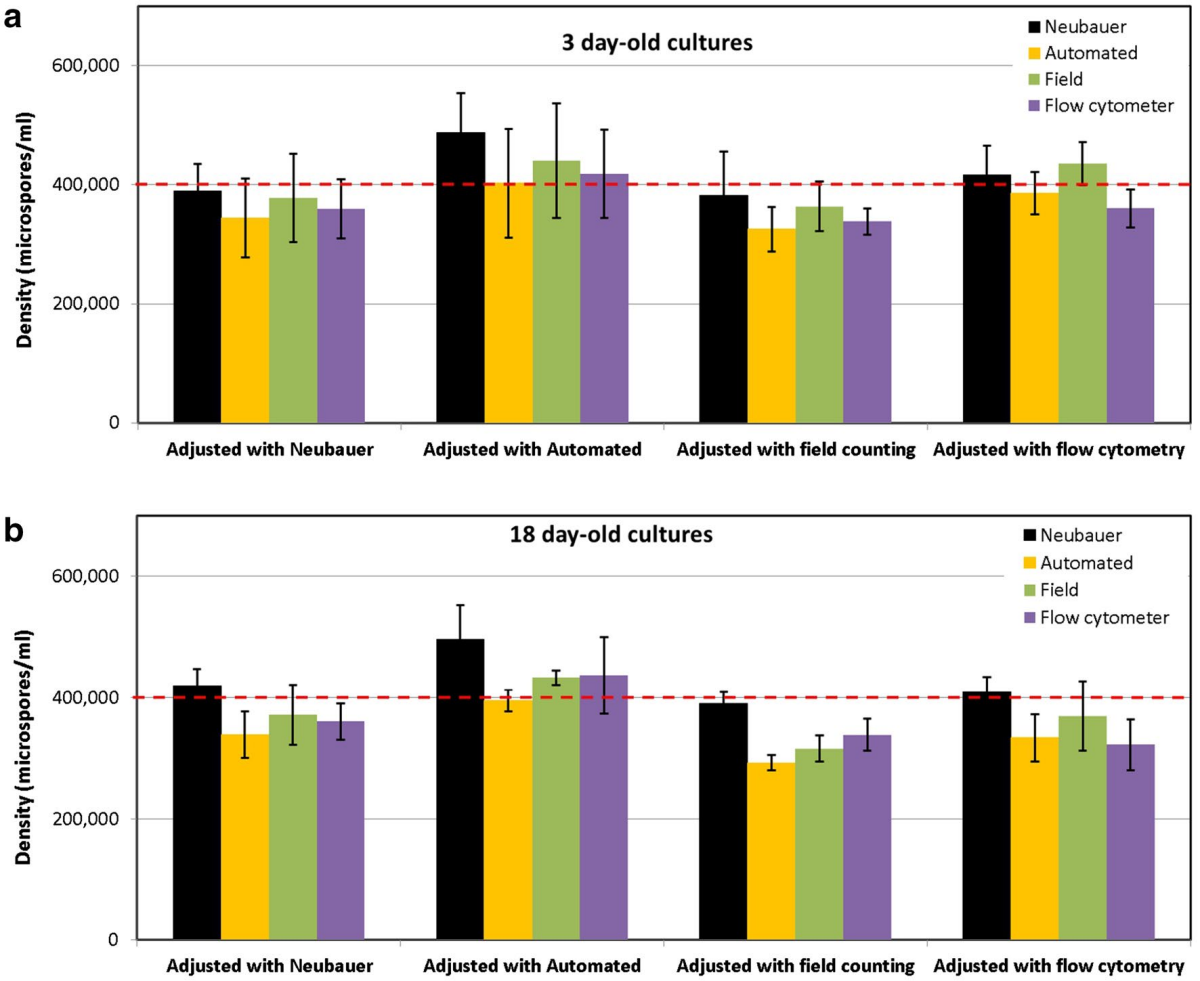
Comparison of microspore density measurements performed at days 3 (**a**), 18 (**b**) with four counting methods in cultures whose initial microspore density was adjusted to 400.000 microspores/ml (red dashed line) with each method. See text for further details

The results presented above were based on microspore cultures whose density was initially adjusted to 400,000 microspores/ml using the Neubauer method. All these results revealed a positive bias of the Neubauer method with respect to most of the other methods (Fig. 4). In order to double check this surprising observation, we performed 12 new cultures and adjusted their initial densities using the Neubauer, cell counter, field and FC + PI counting methods (3 cultures adjusted with each method). Since previous results of FC + PI and SSC showed a nearly exact match (Fig. 4j), we omitted the use of SSC for these experiments, assuming practical equivalence of both methods. Then, we checked culture densities at 3 and 18-day old cultures using each of the four methods, as usual. As seen in Fig. 5, an initial adjustment to 400,000 microspores/ml with the Neubauer method made that 3 and 18-day measurements with the same method were notably similar (2.7 and 4.8% deviation), but 9.2–21.1% higher than those of the other methods. When the automated cell counter was used for the initial adjustment, all counts (with the exception of the automated counter) were above 400,000, being notably higher in the case of the Neubauer method (26.8 and 29.1% for 3 and 18-day old cultures, respectively). An initial adjustment with the field counting method resulted in values around 400,000 when counted with the Neubauer method, but 8.6–21.1% lower when counted with the other three methods, including the initial (field counting). When cultures were initially adjusted with the flow cytometer, counts at days 3 with all four methods revolved around 400,000, but they were clearly below at days 18, except for the Neubauer method. Nevertheless, a tendency to underestimate its own initial count was found for flow cytometry at both timepoints. From these results, we could conclude that the automated counter, field counting and flow cytometry tended to underestimate densities, since when they were used to adjust the initial density, all other methods yielded higher counts at both timepoints. This was specially dramatic in the case of flow cytometry and field counting, which at days 3 and 18 yielded counts lower than in the initial adjustment made using the same methods. As to the Neubauer method, it could be thought that it tends to overestimate densities, since in general, this was the method yielding highest counts. However, it must be noted that in most cases, the Neubauer method showed the counts closest to the expected value of 400,000 microspores/ml.

### Microspore density estimation from fluorosphere counting in mixed samples

The common use of fluorospheres to estimate cell densities implies their use as internal standards mixed with cell suspensions, usually for flow cytometry. In our study, we also tried to estimate microspore density from a known quantity of fluorospheres mixed with cells. Measurements were performed with Neubauer, automated counter, field counting and SSC method. FC + PI method was omitted because of its similarity with SSC. Results of these assays are shown in Table 5. The automated counter image detection system was unable to differentiate between fluorospheres (smaller) and microspores (larger), so we could not obtain any estimation in this case. For the rest of cases, estimations of microspore density were always above the theoretical value, ranging from 19 to 37% of deviation. Thus, we concluded that at least for microspores, this method, although fast and straightforward, is not accurate enough, at least when used with non flow cytometry methods. Indeed, the best results were obtained with the latter, which is reasonable since this counting strategy has been designed for flow cytometry.

**Table 5:** Microspore densities in 3-day old cultures calculated from fluorosphere counting in mixed samples Values are expressed in thousands of particles/ml

Method	Values
Neubauer	621.9 (205.2)
	36.8%
Flow cytometry + SSC	540.7 (83.2)
	19%
Field counting	540.5 (151.6)
	18.9%
Adjusted density	454.5

Each data set includes in the first row the mean (and SD) and in the second row the percentage of deviation from the adjusted density (shown in the last row)

## Discussion

For most cell culture systems, optimal cell progression depends on the optimization of the initial cell density at the onset of the culture. A paradigmatic example of this is isolated microspore culture, where the developmental switch relies on the successful optimization of many different experimental parameters that critically affect the efficiency of the process, and one of them is the density at which microspores are suspended in liquid medium. It affects not only the efficiency of the induction of microspores towards embryogenesis, but also a successful conversion of induced microspores into viable, germinating embryos [3, 5, 10, 16, 37, 38]. Due to the importance of this initial step, not only for microspore culture but for virtually all animal and plant cell cultures, in this work we compared the use of different methods that have been used or could potentially be used to calculate particle densities using two different particles: eggplant microspores and fluorospheres. In light of our results, we can divide the methods used in three groups: flow cytometry methods, automated counter and field counting, and Neubauer chamber. Each has both positive and negative aspects. They are summarized in Table 6 and discussed below.

**Table 6:** Comparison of the methods used to calculate microspore density. Accuracy is expressed in terms of deviation from the real/inicial value, precision in terms of coefficient of variation (CV) and reproducibility in terms of coefficient of repeatability (CR)

	Accuracy (% deviation)	Precision (CV)	Reproducibility (CR)	Approximate time needed (min)	Ease of use	Estimated round basic price	Strength	Weakness
Neubauer	High (13%)	High (17%)	Moderate (175.9)	45	Very easy	€ 260 + microscope	Cheap, straightforward and accurate	Only good precision with high number of countings
Flow cytometry + PI	Low (32%)	Low (26%)	High (39.2)	90^a^	Moderate		Highly reproducible	Slow, expensive, low accuracy and precision
						~€35.000 (optional ~ €29.000)		
Flow cytometry + SSC	Low (30%)	Low (27%)	High (36.3)	15	Easy	~€35.000	Fast, highly reproducible, straightforward	Expensive, low accuracy and precision
Automated counter	Low (32%)	Moderate (19%)	Moderate (109.6)	15	Very easy	€15.000–€20.000	Fast and easy	Moderately expensive and accurate
Field counting	Low (33%)	Moderate (21%)	Low (248.4)	30	Easy	Inverted microscope	Very cheap and straightforward	Laborious, low accuracy and reproducibility

Estimated round prices are given for a Partec Cyflow cytometer excluding taxes

^a^Including the duration of incubation with PI

### Flow cytometry methods are the most reproducible, but they have low accuracy and precision

We evaluated three different methods based on the use of flow cytometry. The first method consisted on the analysis of unstained microspores, assuming that the natural autofluorescence of the exine coat could be sufficiently high to be detected and quantified by the system. However, the analysis of seven 3-day old cultures was enough to realize that this method presented serious limitations. It seemed that exine autofluorescence is not sufficiently intense and/or homogeneous to be detected in all the microspores, at least in our eggplant microspore cultures. Obviously, we strongly discourage its use.

The FC + PI method, together with SSC, provided the most reproducible results, despite the different physical principles used to identify and count flowing microspores. FC + PI and SSC exhibited almost identical results in all the experiments and statistical tests performed. Nevertheless, evaluation of accuracy (using a standard with a known concentration) and precision (with a high number of measurements) lead us to conclude that cytometry methods are not as accurate and precise as the rest. In addition, they repeatedly showed a negative bias with respect to other methods, specially the Neubauer method. Thus, a question arises as to why the flow cytometry methods we used have such a low performance. The use of fluorescent beads is a well-known method to calculate cell densities through flow cytometry, at least for animal cell cultures [39, 40]. Our work is not the only one showing a consistent bias (either positive or negative) of the Neubauer method compared to others [41]. However, other studies have compared hemacytometers versus automated counting methods in animal cells [12, 39, 40, 42–45], and no significant bias has been reported. After a thorough study of the different user-based technical factors that could potentially cause such a bias (including bad calculations, wrong chamber dimensions, pipetting errors, uneven cell distribution, contamination, user-to-user variation, and filling problems, among others), we found a possible cause that might explain such a bias. Several studies comparing methods to calculate the flow rate of flow cytometer fluidics systems, have acknowledged the limitations of many flow cytometers to perform a proper estimation of the volume loaded [46, 47], which may preclude a right estimation of particle densities. This led us to evaluate the volume estimation accuracy of our flow cytometer and, as expected, the effective volume loaded never coincided with the volume estimated by the device (data now shown). This could surely explain the negative bias and the low accuracy and precision of the flow cytometry-based methods we used.

On the other hand, flow cytometers are expensive systems, even in their basic, compact versions. As to FC + PI, a CyFlow cytometer equipped with a Nd-YAG green laser for PI is around €35,000. It is possible to use other fluorescent stains excitable by UV LED light sources, which are cheaper than Nd-YAG green lasers. For example, the CellTracker Blue CMAC Dye from Molecular Probes. However, although UV LED light sources could drop the price of this system down to ~ €29,000, this alternative may still be expensive for many research groups. Another limitation of this method is the need for staining. We used 1 h to ensure a complete and reproducible PI staining. Possibly, this time could be optimized trying different combinations of incubation time and PI concentration, or even other dyes. Anyway, a staining step will always be needed, which may slow down the whole process considerably.

The third flow cytometry-based method tested was SSC analysis. Although we performed this analysis with PI-stained samples, the physics of this method allows for an estimation of the internal complexity of individual particles independent of their fluorescent emission. This means that the time-consuming staining step could be avoided, needing only few min to analyze tens of thousands cells. However, it must be noted that the basic equipment needed to perform this type of analysis has an estimated round cost revolving around €35,000. A similar model with two light sources (a Nd-YAG green laser + a UV LED lamp) may be around €40,000. Clearly, this equipment may not be routinely available for all cell culture laboratories. Alternatively, the user might carry culture vessels to a flow cytometry facility. However, this would imply long times and potential risks for cultured cells, perhaps incompatible with routine cell culturing. Clearly, we discourage this method for that purpose.

### Automated counter and field counting methods have problems associated with image acquisition

The second group of methods tested was field and automatic counting. The working principle of both methods is similar, but as a difference, the field counting method needs only a microscope, and is largely based on an interaction with the user, who must acquire the images or observe microscopic fields, and then count all the particles observed. In principle, it could be thought that a human eye would be more accurate than a machine for cell identification and counting. The different comparisons of the automated counter with field counting revealed that in general, results are very similar between them, which indicates that automated image analysis is at least as correct as human observations. In addition, user-based methods use to be time-consuming and subjected to user bias and putative lack of expertise. Therefore, we postulate that, despite their reduced price and ease of use, the methods that imply a higher user interaction should be avoided in order to increase accuracy and reduce the experimental variation between cultures.

On the other hand, these two methods showed a low accuracy and a moderate precision (see Table 6). In our experience, we have detected some image acquisition issues that might explain it. First of all, we have consistently observed that cells and fluorospheres do not distribute homogeneously in the culture dish, which may make mandatory a software-based tool to compensate for such uneven distribution. The second reason could be automatic focusing, which is common to most image-based automatic counting devices. The algorithm of the equipment we used searches the most contrasted area in the z-axis and takes pictures of it. The problem appears when not all particles are well focused or they are in different focal planes, which precludes their proper identification. Moreover, in the case of fluorospheres, suspended in a viscous solution, long times are needed for them to settle down. In some cases, they do not even settle down, and stay floating. Summarizing, automatic and manual counting methods may not be the best choice to estimate cell or particle suspension densities because they are prone to miss particles out of the focal plane.

The estimated price of the automated counter we used is around €15,000–€20,000. However, it must be noted that it includes a built-in microscope. Other, basic versions of this system need a microscope to be coupled to, but they are much cheaper, which makes it more convenient when the laboratory is already equipped with a light microscope. Once installed and calibrated, it is a rather straightforward and easy-to-use system that allows for quick measurements of cell densities. Other systems such as the Cellometer Auto T4 (Nexcelom Biosciences) have a built-in CCD chip in order to load, image and analyze the sample in the same machine, or use non-image-based methods to count cells, such as the Scepter Cell Counter from Millipore, which uses the impedance-based Coulter principle to detect cells. This avoids the need for a microscope, but for some microscope-independent systems, the cost is similar to that of a microscope + a microscope-coupled automated counter. In addition, Coulter-based systems are applicable only to a limited range of particle sizes. All this considered, automated cell counters appear as a more affordable alternative to flow cytometers.

### The Neubauer chamber showed the best overall performance

As mentioned in the introduction, the methods based on the use of counting chambers appear as the most popular and widely used to calculate cell densities. Most likely, this is due to its affordability. Indeed, this method requires only a Neubauer chamber (around €260) and a basic microscope, available in most laboratories. There may also be a general assumption that, since the use of these methods is widely extended, they must be sufficiently well known and therefore, accurate and reliable. After many different experiments, using both microspores and fluorospheres, the Neubauer method repeatedly showed a positive bias with respect to the other methods used, but its means were always near the theoretical value, whereas the other methods were always below. In addition, analysis of accuracy and precision demonstrated that it is the most reliable method on the basis of its low dispersion and high accuracy. However, it must be noted that the number of cells counted in this work is much higher than that of common routine counts, which surely compensated for the very different results we observed in individual data from each chamber grid (data not shown). Due to this, a major limitation of this method might be the reduced number of cells counted in routine procedures. However, it can be easily overcome by increasing the amount of counted particles.

## Conclusions

Based on our results, it seems evident that among the methods used, those based on flow cytometry are, by far, the most reproducible and concordant, but the worst in terms of accuracy and precision, likely due to an improvable flow rate measurement. Automatic counter and field counting methods showed a low accuracy but a moderate precision. Their actual problem relates to the image acquisition system, improvable too. Perhaps the most important conclusion of this work is that counting chambers and in particular Improved Neubauer chambers are the most reasonable option to routinely measure cell densities. This is important since their use is widely extended among the research community, but there are not abundant comprehensive comparisons to support its use from a technical perspective. In most cases, the reason for this adoption has been “because it was used previously in the protocol we are applying”. Nevertheless, our advice to future users of Neubauer chambers would be to increase the number of cells counted in each assay, in order to reach these standards of accuracy and low dispersion.

## Additional files

**Additional file 1: Fig. S1.** Improved Neubauer chamber showing the different large and small grids.

**Additional file 2: Table S1.** Definition of the different parameters used in this article based on ISO normative.
