# Supplementary figures and images for: Comparison of six different methods to calculate cell densities
Source: Plant Methods. 2018 Apr 16;14:30. doi: 10.1186/s13007-018-0297-4 (PMC5901878; doi:10.1186/s13007-018-0297-4)

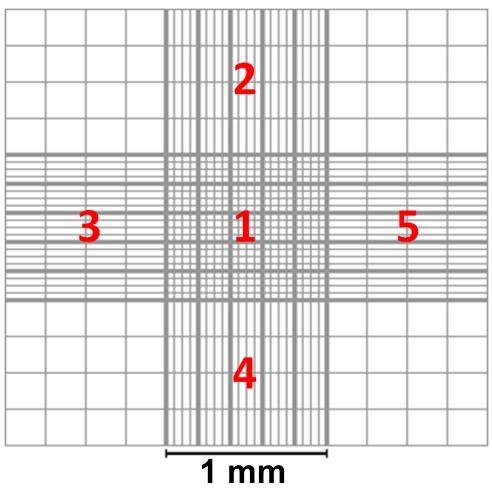

Supplement: Supplementary file 1 — Additional file 1: Fig. S1. Improved Neubauer chamber showing the different large and small grids. [file 13007_2018_297_MOESM1_ESM.jpg]
