# Supplementary material for: Comparison of six different methods to calculate cell densities
Source: Plant Methods. 2018 Apr 16;14:30. doi: 10.1186/s13007-018-0297-4 (PMC5901878; doi:10.1186/s13007-018-0297-4)
Supplement: Supplementary file 2 — Additional file 2: Table S1. Definition of the different parameters used in this article based on ISO normative. [file 13007_2018_297_MOESM2_ESM.docx]

**Additional file 2: Table S1:** definition of the different parameters used in this article based on ISO normative.

| **Accuracy** | The closeness of agreement between a test result and the accepted reference value. |
| --- | --- |
| **Bias** | The difference between the expectation of the test results and an accepted reference value. |
| **Precision** | The closeness of agreement between independent test results obtained under stipulated conditions. |
| **Reproducibility** | Precision under different conditions. |
